# Healthcare professionals' intentions to use wiki-based reminders to promote best practices in trauma care: a survey protocol

**DOI:** 10.1186/1748-5908-5-45

**Published:** 2010-06-11

**Authors:** Patrick M Archambault, France Légaré, André Lavoie, Marie-Pierre Gagnon, Jean Lapointe, Sylvie St-Jacques, Julien Poitras, Karine Aubin, Sylvain Croteau, Martin Pham-Dinh

**Affiliations:** 1 Centre hospitalier affilié universitaire Hôtel-Dieu de Lévis, 143, rue Wolfe, Lévis, G6V3Z1, Canada; 2 Centre de recherche du Centre hospitalier universitaire de Québec (CRCHUQ), 10, rue de l'Espinay, Québec, G1L 3L5, Canada; 3 Centre de recherche FRSQ du CHA universitaire de Québec, 1401, 18e Rue, Québec, G1J 1Z4, Canada; 4 Faculté des sciences infirmières, Pavillon Ferdinand-Vandry, 1050, avenue de la Médecine, Local 3645, Université Laval, Québec, G1V 0A6, Canada; 5 Agence d'évaluation des technologies et des modes d'intervention en santé (AÉTMIS), 2021 avenue Union, bureau 1040, Montréal, H3A 2S9, Canada; 6 Institut national de santé publique, 945, avenue Wolfe, Québec, G1V 5B3, Canada; 7 Hôpital de Gatineau, 909 Verendrye Ouest, Gatineau, J8P 7H2, Canada

## Abstract

**Background:**

Healthcare professionals are increasingly using wikis as collaborative tools to create, synthesize, share, and disseminate knowledge in healthcare. Because wikis depend on collaborators to keep content up-to-date, healthcare professionals who use wikis must adopt behaviors that foster this collaboration. This protocol describes the methods we will use to develop and test the metrological qualities of a questionnaire that will assess healthcare professionals' intentions and the determinants of those intentions to use wiki-based reminders that promote best practices in trauma care.

**Methods:**

Using the Theory of Planned Behavior, we will conduct semi-structured interviews of healthcare professionals to identify salient beliefs that may affect their future use of wikis. These beliefs will inform our questionnaire on intended behavior. A test-retest of the survey will verify the questionnaire's stability over time. We will interview 50 healthcare professionals (25 physicians and 25 allied health professionals) working in the emergency departments of three trauma centers in Quebec, Canada. We will analyze the content of the interviews and construct and pilot a questionnaire. We will then test the revised questionnaire with 30 healthcare professionals (15 physicians and 15 allied health professionals) and retest it two weeks later. We will assess the internal consistency of the questionnaire constructs using Cronbach's alpha coefficients and determine their stability with the intra-class correlation (ICC).

**Discussion:**

To our knowledge, this study will be the first to develop and test a theory-based survey that measures healthcare professionals' intentions to use a wiki-based intervention. This study will identify professionals' salient beliefs qualitatively and will quantify the psychometric capacities of the questionnaire based on those beliefs.

## Background

Clinical practice does not always reflect best evidence, and high proportions of inappropriate care have been reported in different healthcare systems and settings [1]. Inappropriate care significantly impacts patient outcomes and healthcare costs. In emergency departments, unconscious acts of omission and information overload [2] contribute to inappropriate care. Systematic reviews have indicated that reminders to healthcare professionals can be effective in promoting change in healthcare professionals' practices in a variety of clinical areas and environments [3-6]. These reminders can take the form of protocols with check boxes, admission order sets, care maps, clinical decision rules, patient handouts, or decision aids. To increase professionals' use of best practices, reminders must be based on evidence and clinical practice guidelines. As the rate of new evidence accelerates [7], however, updating reminders becomes more difficult. Furthermore, new reminders promoting best practices are difficult to implement rapidly, as numerous stakeholders must approve the changes. These stakeholders--who include physicians, registered nurses, respiratory therapists, pharmacists, hospital administrators, and patients-- often review the changes in committees.

In emergency departments, both time and collaborative partnerships within and across care teams are important factors in the creation, use, and updating of reminders that promote best practices [8,9]. Convincing stakeholders to use, update, and create new reminders promoting best practices can be a difficult task in emergency departments, where shift work is prevalent. In this context, a wiki could be a powerful tool that permits stakeholders from a single or many emergency departments to collaborate asynchronously in the updating and creation of reminders while avoiding the duplication of efforts and minimizing time investments.

A wiki is a web page or collection of web pages whose content can be modified by those who access it. As such, a wiki can easily become a common repository of information for stakeholders working in different emergency departments [10-12]. A wiki can function as a tool that facilitates different phases of the knowledge-to-action cycle [13], and act as a 'virtual agora' where stakeholders from different professions and settings can share, update, and create reminders that promote best practices. For example, wikis are fast becoming an important tool of mass collaboration that helps science harness thinking across the world to map the human genome (WikiGenes [14]). Wikis are also being used to promote the sharing of information, know-how, and wisdom among researchers and clinicians working in medicine [11,15-17]. Clinicians have demonstrated great interest in Web 2.0 collaborative tools for medical education [18], but for any wiki to work as a collaborative tool, users must contribute actively to its content. In order to develop a wiki that helps healthcare professionals implement best practices in the emergency department, the stakeholders must adopt specific behaviors. Our research project aims to develop a validated questionnaire to assess stakeholders' intention to adopt one of these behaviors.

### Clinical context of this study

Adherence to clinical practice guidelines in caring for traumatic brain injury victims has decreased mortality, morbidity, and the cost of care in the United States and Europe [19-27]. In the United States, traumatic brain injury is the leading cause of death and disability in children and adults aged 1 to 44 [28]. Every year, approximately 52,000 deaths occur from traumatic brain injuries [28]. Traumatic brain injury hospitalization rates have increased from 79 per 100,000 in 2002 to 87.9 per 100,000 in 2003 [29].

Given the tight time constraints associated with traumatic brain injuries, healthcare professionals who care for traumatic brain injury victims must make a series of decisions under great pressure. For example, the physician must select an induction agent to intubate a severe traumatic brain injury victim [30-32]; decide whether the patient needs a computed tomography (CT) scan [33-35]; and choose treatment for intracranial hypertension [36]. Reminders promoting best practices could help inform these decisions [37] and increase healthcare professionals' adherence to clinical practice guidelines. But these reminders must be updated whenever new evidence or new clinical practice guidelines become available [38].

According to a survey of trauma coordinators and nurse managers caring for traumatic brain injury victims in the United States, adherence to clinical practice guidelines has improved in level I trauma centers since the introduction of the Brain Trauma Foundation clinical practice guidelines [39]. However, information concerning adherence to traumatic brain injury clinical practice guidelines in other countries and in level II and III trauma centers is lacking. Ongoing research will help fill this gap in the knowledge [40], but there is no reason to believe that adherence to traumatic brain injury clinical practice guidelines worldwide is better than adherence reported in the United States. Our study hypothesizes that a wiki devoted to supplying healthcare professionals with easy access to reminders and allowing healthcare professionals to update those reminders rapidly would improve healthcare professionals' endorsement of clinical practice guidelines and help them translate the guidelines into practice. Because successful exploitation of a wiki depends on healthcare professionals' adoption of specific behaviors, we begin by assessing healthcare professionals' intention to adopt these behaviors.

### Conceptual underpinnings of the proposed study

**Figure 1:**
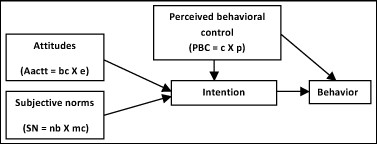
**Theoretical framework of the Theory of Planned Behavior**.[41]

The Theory of Planned Behavior (TPB) [41] (Figure 1) is well known for its application to the study of healthcare professionals' behaviors [42-49]. TPB provides a theoretical account of the ways in which attitudes, subjective norms, and perceived behavioral control combine to predict behavioral intention [50]. It postulates that when an individual has some control over a situation, intention is the immediate determinant of behavior [42].

Intentions are influenced by three constructs: attitudes, subjective norms, and perceived behavioral control. Attitudes ('Aact' in Figure 1) are defined as the actor's beliefs about the consequences (the advantages and disadvantages) of a behavior. Attitude is assumed to have two interacting components: beliefs about the consequences of a behavior ('bc' in Figure 1), and judgments--positive or negative--about each feature of the behavior (outcome evaluation or 'e' in Figure 1). Subjective norms ('SN' in Figure 1) refer to perceived social pressure to engage or not to engage in a behavior. Subjective norms are also assumed to have two interacting components: beliefs about how people who are in some way important to the actor would like the actor to behave (normative beliefs or 'nb' in Figure 1), and the actor's positive or negative judgments about each belief (motivation to comply or 'mc' in Figure 1).

Perceived behavioral control reflects an actor's perception of how difficult it is to perform a given behavior. This perception is determined by control beliefs ('c') about the power of situational and internal factors to inhibit or facilitate the actor's performance of the behavior (perceived power to influence, or 'p' in Figure 1).

### Objectives

Our goal is to survey healthcare professionals' intentions to use a wiki-based reminder that promotes best practices for the management of severe traumatic brain injury victims in emergency departments in the province of Quebec, Canada. This behavior is described in detail in Appendix 1.

Our specific objectives are to identify healthcare professionals' salient beliefs about attitudes, social norms and perceived behavioral controls regarding the use of a wiki-based reminder that promotes best practices for the management of severe traumatic brain injury victims in emergency departments in the province of Quebec, Canada; and to test the metrological properties of a new questionnaire on this topic.

## Methods

### Study design

**Figure 2:**
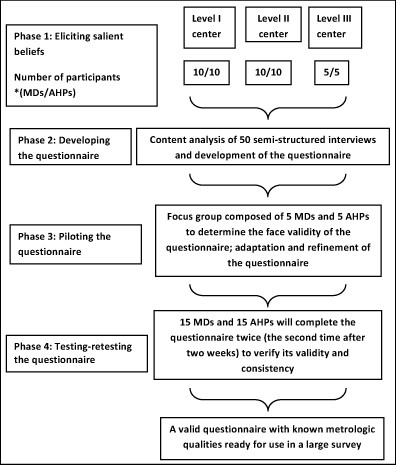
**Flow chart of the phases of the development of the questionnaire**.

This study has four phases (Figure 2): eliciting healthcare professionals' salient beliefs by conducting a cross-sectional qualitative study of beliefs related to the behavior defined in Appendix 1 using semi-structured interviews; developing the questionnaire; piloting the questionnaire; and testing-retesting the questionnaire.

### Phase one: Eliciting salient beliefs

### Participants

The study will take place in three officially designated trauma centers in the province of Quebec, Canada: a level I, a level II, and a level III trauma center. All 59 of Quebec's designated trauma centers have structured trauma committees whose oversight of the quality of care administered to injured patients is required for their designation. These committees already comprise various actors involved in the care of trauma patients: emergency physicians, emergency nurses, surgeons, and hospital administrators. In level I centers, the trauma committee also includes intensivists, neurosurgeons, and imaging and rehabilitation professionals. The provincial government has expressed its desire to standardize the care offered by Quebec's trauma centers. If care does not reach certain standards, underperforming centers may lose their designation. Considering this impetus to improve the standard of care, we resolved to assess stakeholders' intentions to use a wiki-based reminder that promotes best practices in the management of traumatic brain injury victims.

Our study will involve two types of healthcare professionals: physicians (excluding residents and medical students) and allied health professionals (excluding trainees and students) such as registered nurses, pharmacists, respiratory technicians, social workers, physiotherapists, and other members of local trauma committees involved in the care and the planning of care for trauma patients. These healthcare professionals will be asked to participate in a semi-structured interview. Godin and Kok [51] have determined that a sample of 25 participants is sufficient to elicit salient beliefs in an elicitation study. Accordingly, interviewing a minimum of 25 physicians and 25 allied health professionals from three healthcare centers will permit us to respect the theoretical framework of this study for each group of healthcare professionals.

After obtaining participants' consent, research assistants will conduct individual semi-structured interviews with the help of a written clinical vignette and a video that demonstrates the behavior of interest. We will conduct our interviews in the emergency departments of three hospital trauma centers. The first hospital is a level II trauma center with orthopaedic surgery and general surgery support. The second hospital is a level I trauma center that offers the full scope of definitive care, including neurosurgery. The third hospital is a level III trauma center with surgical and orthopaedic support. We will individually survey 10 physicians and 10 allied health professionals from the level II center, 10 physicians and 10 allied health professionals from the level I center, and five physicians and five allied health professionals from the level III center.

### Data collection procedure

First, we will write a clinical vignette with the help of three clinical experts, two of whom will be members of Quebec's trauma center accreditation board. The vignette will address the behavior of interest in a typical case of severe traumatic brain injury experienced in an emergency department in the province of Quebec. Two medical informatics experts will ensure that the vignette describes the wiki-based reminder being incorporated into daily practice. We will then videotape the vignette, using actors.

All survey participants will watch the same video and read the same clinical vignette. After watching the video and reading the vignette, the participants will be interviewed by a research assistant, who will use a semi-structured questionnaire. Interviews will be digitally recorded and transferred to a computer for future reference. The interviewer will note participants' answers on paper forms that correspond to the interview format. All participants will remain anonymous.

The semi-structured interviews will elicit participants' feedback concerning the following elements: the advantages and disadvantages of adopting the defined behavior; influential people who would approve or disapprove of the behavior; and barriers and facilitators of the behavior.

### Content analysis

Two independent research assistants will analyze the content of the recorded interviews and their written summaries to identify participants' salient beliefs. They will classify responses into themes (salient beliefs) and through discussion, decide how to label the themes. Themes that express the same idea will be grouped and their frequency calculated. The themes will then be ordered from the most to the least frequently mentioned. All themes will be assigned a number that corresponds to the questionnaire in which the theme was identified. Within each theme, beliefs will be compared to determine whether they are unique. The research assistants will then produce a single list of salient beliefs for each construct. Any dissent between research assistants will be resolved by the principal investigator, who will make the final decision.

To assess the attitudinal construct, the interviews will elicit respondents' perceptions of the advantages and disadvantages of using wiki-based reminders. The research assistants will group these advantages and disadvantages into themes (behavioral beliefs), which they will rank from the most to the least frequently mentioned.

For the subjective norm construct, the interviews will identify groups, organizations, and categories of individuals (reference groups) likely to apply social pressure with respect to the two defined behaviors. The research assistants will group these sources of social pressure into themes (normative beliefs), label the themes, and rank them from the most to the least frequently mentioned.

Finally, to assess perceived behavioral control, the research assistants will analyze the content of the interviews and classify the information into themes (control beliefs), and label and order them just as for the other constructs.

### Phase two: Developing the questionnaire

We will base our questionnaire format on a document that describes the construction of a TPB-based survey [52]. We will measure the 'intention' construct directly, and the following constructs both directly and indirectly: 'attitudes,' 'subjective norms,' and 'perceived behavior control.' We will measure intention using the generalized intention method described by Francis *et al*. [52]. To achieve adequate coverage of our target population, in measuring each construct, we will retain the top 75% of beliefs (behavioral, normative, and control) most frequently occurring in the content analysis of the interviews. The following four sections describe how we will measure constructs indirectly and list the healthcare professional characteristics that we will assess.

### Attitude (Aact) construct questions

We will convert the top 75% behavioral beliefs (b) most frequently occurring in the content analysis into a set of statements that reflect beliefs that might affect the behavior of our target population. Each belief statement will be converted into an incomplete sentence. By completing the sentence using a set response format such as 'extremely undesirable to extremely desirable,' the participant will evaluate the statement either positively or negatively (outcome evaluation or e).

### Subjective norm (SN) construct questions

We will convert the top 75% reference groups or individuals most frequently occurring in the content analysis into the 'stems' of normative belief (nb) items. We will then construct questionnaire items to assess the strength of normative beliefs with respect to each reference group, conceiving the findings as motivation to comply (mc) with pressure from each group. We will assess motivation to comply using a standardized format for all assessments. Items will reflect what important people think a person should do (injunctive norms) and what important people actually do (descriptive norms). For each source of social pressure, we will write a statement about the importance of that source. By responding to the statements, participants will indicate the strength of their motivation to comply with the values of each reference group or individual.

### Perceived behavioral control (PBC) construct questions

We will convert the top 75% of most frequently occurring control beliefs into statements that reflect the beliefs that might make it difficult for the participant to perform (or not perform) the target behaviors. To assess the influence of these factors on participants' behavior, we will convert each control belief (c) statement into an incomplete statement about whether the belief makes it more or less likely that the participant will perform the target behavior, or whether the belief makes the behavior easier or more difficult to perform (perceived power to influence, or p).

### Characteristics of healthcare professionals

To assess the impact of healthcare professionals' attributes on their behavioral intention to consult the wiki-based reminder, we will assess the following characteristics: age, gender, type of healthcare professional and diploma, emergency physicians' level of training, type of healthcare center (level I, level II, or level III trauma center) where the healthcare professional works, number of years of practice, presence of computers with unrestricted access to internet within the emergency department, previous consultation or contribution to a wiki, membership in a local trauma committee, and number of traumatic brain injury victims treated in the last year.

### Questionnaire format Number and content of questions

The first draft of the questionnaire will include:

1. Questions that elicit demographic information about the healthcare professional respondent.

2. Questions regarding the defined behavior:

2a. Questions developed during the elicitation phase for the six indirectly measured constructs: behavioral beliefs (b), outcome evaluation (e), normative beliefs (nb), motivation to comply (mc), control beliefs (c), and perceived power to influence (p). The number of questions will depend on the number of salient beliefs retained.

2b. Questions that directly measure the constructs identified in our theoretical model (three questions for each construct): intention, perceived behavioral control, attitude, and subjective norm.

We estimate approximately six salient beliefs for the defined behavior. Accordingly, with 36 indirect items and 12 direct items, the questionnaire will comprise 48 carefully worded items that assess all the constructs related to the behavior of study. It will also comprise 10 questions about healthcare professionals' characteristics.

### Ordering of questions

Items relative to different constructs will be mixed throughout the document. That is, questions used to measure intention will be interspersed with questions measuring attitudes, subjective norms, and perceived behavioral control.

### Phase three: Pilot-testing the questionnaire

We will pilot-test our questionnaire by asking a focus group of 10 participants (five physicians and five allied health professionals) from our sample population to answer the questionnaire and tell us whether they had difficulty answering it. We will compare two methods of administering the questionnaire: a paper method and a web method (SurveyMonkey: www.surveymonkey.com). Five focus group volunteers will answer a paper survey and the other five will answer a web survey. We will check comprehension and clarity for both surveys. If necessary, we will modify the wording of the questions. To accomplish this, pilot-test participants will be asked to: read the instructions and tell us what they understand; state what our questions mean to them; identify ambiguous or complex terms; specify their ease or difficulty in answering our questions and discuss any difficulties; identify the most difficult questions; specify whether each answer option is reasonably different from the others and if not, identify options that are too similar; and suggest changes to answer options that are too ambiguous or that do not adequately express their opinions. In addition, we will assess how the length of the questionnaire affects participant fatigue and response rates. If the length of the questionnaire decreases the response rate, we will consider reducing the number of items measured or even forego measuring constructs that do not substantially help explain variances in behavioral intention. Finally, we will compare the time required to take the web survey versus the paper survey. We will also assess participants' preference for the web or the paper survey.

### Phase four: Test and retest at two weeks

After making adjustments in the pilot phase, we will test the revised questionnaire with at least 30 participants with similar characteristics as the target population (15 physicians and 15 allied health professionals). These participants will not have participated in the elicitation phase. The same questionnaire will be re-tested two weeks later with the same 30 participants. Half the group will be asked to volunteer to answer the online questionnaire; the other half will answer the paper questionnaire. This second test will permit us to assess: respondents' compliance with instructions; respondents' reactions to certain items and words; any hesitations or questions on the part of respondents; and participants' preference for a web versus a paper survey. This information will be valuable when we interpret test results with regard to the time required to complete the questionnaire, the variability in answers for each item (so that we exclude items that fail to discriminate), and the links between items. Determining participants' preference for a web versus a paper survey will help us decide how to conduct the survey provincewide.

### Data analysis of the questionnaire's metrologic characteristics

We will measure the internal consistency of the constructs (the tendency of answers within a group of constructs) using Cronbach's alpha coefficients. To measure the stability of constructs over time, we will measure an adjusted agreement intra-class correlation coefficient (ICC). We will perform statistical analyses using SAS version 9.1.3 (SAS Institute Inc., Cary, NC).

## Discussion

To our knowledge, this study will be the first to develop and test a theory-based questionnaire that surveys healthcare professionals' intentions to use a wiki-based intervention in the emergency department. The study will identify behavioral salient beliefs qualitatively and will quantify the psychometric capacities of a questionnaire based on those beliefs. Our findings will allow us to determine which salient beliefs are the most important to retain in a questionnaire that will survey a broader stakeholder population with regard to stakeholders' consultation of a wiki about evidence-based protocols for traumatic brain injury care in the emergency department.

To the best of our knowledge, this study will also be one of the first to assess healthcare professionals' intention to adopt a complex behavior (defined as a set of smaller behaviors) by using a video that depicts the small, implicit, lead-in behaviors necessary to perform the behavior in question: logging onto the Internet, using a keyboard to type the search terms necessary to find the wiki-based reminder, printing the wiki-based reminder, choosing which of the prescriptions suggested by the wiki-based reminder to prescribe, adding the wiki-based reminder to the medical chart, and persuading nursing personnel to administer the prescriptions selected. Other studies have used theory-based clinical vignettes to assess participants' intention to adopt certain behaviors [53,54] and to assess the quality of clinical practice [55]. We believe that using a video in addition to a written vignette will allow us to differentiate the target behavior (using the wiki-based reminder) from the general objective (applying best practices to the care of severe traumatic brain injury victims in Quebec), which objective will not be assessed using the TPB.

In addition, we will develop and validate a paper and a web survey. Only using a web survey could induce bias in our measurement of healthcare professionals' intention to use a web-based tool, because healthcare professionals who are not computer or web-savvy will probably avoid answering the web survey. The results from the pilot and the test-retest phases of our study will allow us to compare healthcare professionals' intentions to use wiki-based reminders in light of their preference of survey method (a paper versus a web survey).

### Potential study limitations and how they will be addressed

Our TPB-based survey will help identify the determinants of allied health professionals' and physicians' intentions to perform the behavior of interest. This behavior is still theoretical and complex, because the tool proposed (the wiki) has not yet been developed. Because the behavior of study requires many smaller, lead-in behaviors, it would be difficult for participants to understand what the behavior truly implies with only a written clinical vignette and a theoretical description of how the wiki would work. This is why we will show participants a video of the wiki and the behavior we wish to study.

If a theory-based intervention developed from the results of this study is unsuccessful in increasing healthcare professionals' consultation of a wiki-based, evidence-based reminder, we will re-analyze the determinants of behavioral intention at a more granular level. While we hope to generalize the results of our study to a broader clinical context (settings other than trauma), it is possible that our theory-based intervention will only be valid for the context of this survey.

This study is only the first step in our attempt to understand physicians' and allied health professionals' intentions to consult a wiki for content. It is nonetheless essential, because a wiki requires the collaboration of many users who must adopt certain behaviors. By definition, a wiki is the product of its users and is only relevant as long as users update it and create new content. By understanding the behavioral intentions of potential users (physicians and allied health professionals) to consult the wiki, we can better understand how a wiki could be used as an intervention to increase evidence-based practices.

Time constraints [37,56] are a major barrier to studying clinicians' behavior in the emergency department. Considerations of the length of the questionnaire thus limits the number of behaviors our study can assess. Several other behaviors could be studied and might need to be studied in the future. For example, we will not assess healthcare professionals' intentions to update existing wiki-based reminders and to create new wiki-based reminders. We acknowledge this limitation, but believe that our questionnaire will address the most important behavior at this time. If our findings reveal that clinicians do not intend to use the wiki during the course of fulfilling their clinical duties, it is important that we understand the determinants of this behavior before we ask clinicians to update and create wiki-based reminders.

### Ethical aspects

This study protocol has been approved by the ethics review boards of all three hospitals in the study. All interviewees will remain anonymous, and interviews will be conducted by a research assistant who will not have met respondents prior to interviewing them. Answers will be recorded and numbered so that we can link a given belief to a given interview for future reference and discussion if necessary. Voice recordings will only be audited by the research assistants and the person who transcribes the interviews.

## Competing interests

SC is presently developing a wiki-based decision support tool. There are no financial competing interests related to this tool. This tool will be free like other existing wikis. There are no patents pending for this tool. All other authors declare that they have no competing interests.

## Authors' contributions

The principal investigator (PA) designed and wrote this protocol. FL, AL, MPG, JL, SSJ, JP, KA, SC, and MPD reviewed and modified different versions of this protocol. SC, MPD and PA conceived the idea of the wiki. All authors have read and approved the final manuscript.

## Appendix 1. Definition of the behavior

**Action: **To use

**Target: **a wiki-based reminder promoting best practices

**Context: **for the management of severe traumatic brain injury victims in emergency departments of the province of Quebec, Canada
